# A genome-wide association analysis: m6A-SNP related to the onset of oral ulcers

**DOI:** 10.3389/fimmu.2022.931408

**Published:** 2022-07-25

**Authors:** Zhuoxuan Wu, Weimin Lin, Quan Yuan, Mingyue Lyu

**Affiliations:** ^1^ State Key Laboratory of Oral Diseases & National Clinical Research Center for Oral Diseases, West China School of Stomatology, Sichuan University, Chengdu, China; ^2^ State Key Laboratory of Oral Diseases & National Clinical Research Center for Oral Diseases, Department of Oral Implantology, West China Hospital of Stomatology, Sichuan University, Chengdu, China

**Keywords:** GWAS, m6A, oral ulcers, CCRL2, pathogenesis

## Abstract

Oral ulcers are one of the most common inflammatory diseases on oral mucosa that have obvious impacts on patients. Studies have shown that N6-methyladenosine (m6A) RNA transcription modification may be involved in the development of various inflammatory responses, and whether the pathogenesis of oral ulcers is related to m6A is unclear. This study aims to identify how m6A-related single nucleotide polymorphisms (m6A-SNPs) may affect oral ulcers. The UKBB dataset containing 10,599,054 SNPs was obtained from the GWAS database using the keyword “oral ulcer” and compared with the M6AVar database containing 13,703 m6A-SNPs.With 7,490 m6A-SNPs associated with oral ulcers identified, HaploReg and RegulomeDB were used for further functional validation and differential gene analysis was performed using the GEO database dataset GSE37265. A total of 7490 m6A-SNPs were detected in this study, 11 of which were related to oral ulcers (p<5E-08), and all of these SNPs showed eQTL signals. The SNP rs11266744 (p=2.00E-27) may regulate the expression of the local gene CCRL2, thereby participating in the pathogenesis of oral ulcers. In summary, by analyzing genome-wide association studies, this study showed that m6A modification may be involved in the pathogenesis of oral ulcers and CCRL2 may be the targeted gene.

## Introduction

Oral ulcers are one of the most common inflammatory diseases on oral mucosa, particularly affecting adolescents and young adults (1–3), and the incidence in the general population has reached about 1.8-5% (4–7). Severe aphthous ulcers are difficult to heal, greatly affecting patients’ daily diet and mental state. The causes of oral ulcers may include infection, local trauma, changes in hormone levels, vitamin deficiencies and genetics (8, 9). According to many studies, genetic factors were associated with oral ulcers (10, 11). A questionnaire survey of 684 patients with oral ulcers reported that 66% of patients have a history of oral ulcers in family members (10). Another retrospective study on 1,160 parents and their children showed that the variation of the latent phenotype in the incidence of oral ulcers was caused by an additive genetic factor (64%), a common environmental factor (26%) and a specific environmental factor (10%) (11). In addition to clinical studies, *in vitro* studies also showed that C677T mutations in the MTHFR gene were associated with the development and severity of oral ulcers (12).

N6-methyladenosine (m6A) refers to the methylation of the sixth N atom on the adenine base. As one of the most abundant chemical modifications on mammalian mRNA and non-coding RNA, it is involved in many biological activities including inflammatory responses (13, 14). Oral ulcers are featured in inflammatory responses stimulated by a variety of inflammatory cells, metabolic enzymes and cytokines (15). Studies showed that approximately 0.1% to 0.4% of adenosines in mRNA were modified by m6A, with an average of 2-3 m6A modification sites per transcript (16, 17). By recruiting specific protein complexes at the m6A modification site on RNA, the structure, function and stability of mRNA can be regulated, thereby regulating gene expression or splicing (16, 18). Proteins such as cofactor Wilms tumor 1 related protein (WTAP) (19), obesity-related protein (FTO) (20), and cytoplasmic YTH domain-containing family member 1(YTHDF2) (21) can be involved in the regulation of inflammatory responses by altering m6A modifications (22, 23). And dysregulation of local inflammatory response in the oral mucosa can disrupt normal mucosal structures and induce oral ulcers. In addition, m6A-SNP affects m6A methylation and related biological processes by changing the RNA sequence of the target site or key flanking nucleotides (24).

Based on the current analysis of the m6A-SNP list and published GWAS data, m6A-SNPs were found to be associated with diseases with inflammatory responses such as periodontitis (25), type 2 diabetes (26, 27) and obesity (28). On the other hand, oral ulcers are characterized by inflammatory responses, so it is worthwhile to further explore the relationship between m6A-SNPs and oral ulcers. Genome-wide association studies (GWAS) assess the association between SNPs and traits by analyzing multiple genetic variants in individuals with different phenotypes (29). Previous studies determined oral ulcer susceptibility loci located in the protein-coding region by GWAS, such as coding key cytokines (tumor necrosis factor-α, interleukin-1α, interleukin-10 and interleukin-12) gene region variation (9, 30). In addition to the SNPs mentioned above, a large number of SNPs are located in non-protein-coding regions. Specifically, studies showed that SNPs located in the untranslated region (UTR) could also affect RNA secondary structure or RNA–protein interactions (31). Moreover, these SNPs could also alter enhancers or silencers during exon splicing (32).

Above all, oral ulcer is an inflammatory disease closely associated with genetic factors, and m6A-SNP directly affects gene expression involved in the development of inflammatory responses. Since whether m6A-SNP is involved in the pathogenesis of oral ulcers is still unclear and GWAS help to fully reveal the genes associated with oral ulcers development, exploring how m6A-SNPs related to oral ulcers may provide a new perspective on the genetic mechanism of oral ulcers. Therefore, this study aims to explore the role of m6A-SNP in the occurrence and development of oral ulcers by analyzing the original data of the published oral ulcer GWAS and the list of m6A-SNPs in the M6Avar database.

## Methods

### Identification of oral ulcers‐associated m6A‐SNPs

Firstly, we downloaded the GWAS summary data on oral ulcers in UKBB (the oral ulcer, 39439 cases and 345587 controls). In order to ascertain the m6A-SNPs that might have an effect on the methylation of m6A, we downloaded the list of m6A-SNPs from the publicly available M6AVar database and compared it with the comprehensive statistics of oral ulcers (33). Currently, the M6AVar database contains 13,703 high confidence levels (miCLIP/PA-m6A-seq experiments), 54,222 medium confidence levels (MERIP-Seq experiments), and 245,076 low confidence levels (random based on the random forest algorithm) of human m6A-SNP genome prediction (33). In the following analysis, p<0.05 was designed as the threshold for statistical significance.

### Expression quantitative trait loci analysis of oral ulcers‐associated m6A‐SNPs

One of the ways that m6A modification exerts a biological effect is to affect the regulation of local genes. After identifying the m6A-SNPs associated with oral ulcers, online tools were used to annotate them to explore the mechanism of their biological functions. The HaploReg browser (http://archive.broadinstitute.org/mammals/haploreg/haploreg.php) is used to detect whether the eQTL signal of the identified m6A-SNP is displayed, and functional evidence can also be obtained (p<0.05) (34). In addition, through HaploReg and RegulomeDB (http://regulome.stanford.edu/), functional verification of the identified m6A-SNPs was performed to determine their possible roles in transcriptional regulation.

### Prediction of m6A modification near m6A-SNPs

The m6A-SNPs identified by the analysis above were input into an online m6A modification prediction tool (SRAMP, http://www.cuilab.cn/sramp/) for further analysis. It can determine whether m6A-SNP affects surrounding m6A modifications by analyzing input reference sequences and altered sequences (such as genome sequences or cDNA sequences) (35).

### Differential expression analysis of local genes

To explore the local gene expression of m6A-SNPs in the pathogenesis of oral ulcers, transcriptomic data from the GEO database of ulcerated and normal mucosa were used for differential gene analysis. In the GEO database (http://www.ncbi.nlm.nih.gov/geo), we downloaded the data set GSE37265 containing standardized oral ulcer-related gene expression signals (14 recurrent aphthous ulcer sites tissue and 14 normal controls). For the average gene expression signals of oral ulcer patients and healthy controls, a t-test method was used to analyze the differential expression. The significance level of p<0.05 was used for differential expression analysis.

## Results

### Identification of oral ulcers-associated m6A-SNPs

**Figure 1:**
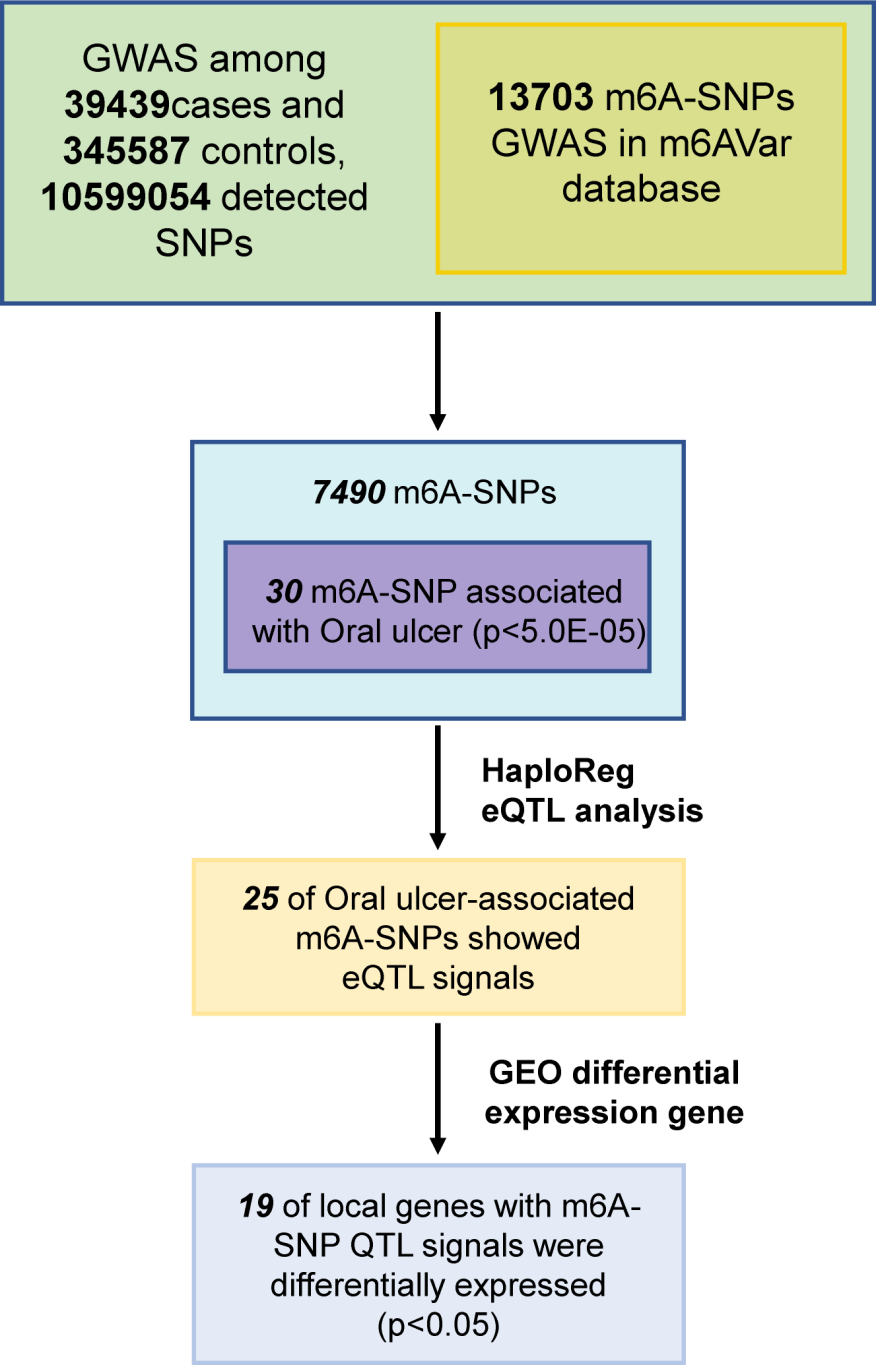
Flow chart of research designs and main results.

**Figure 2:**
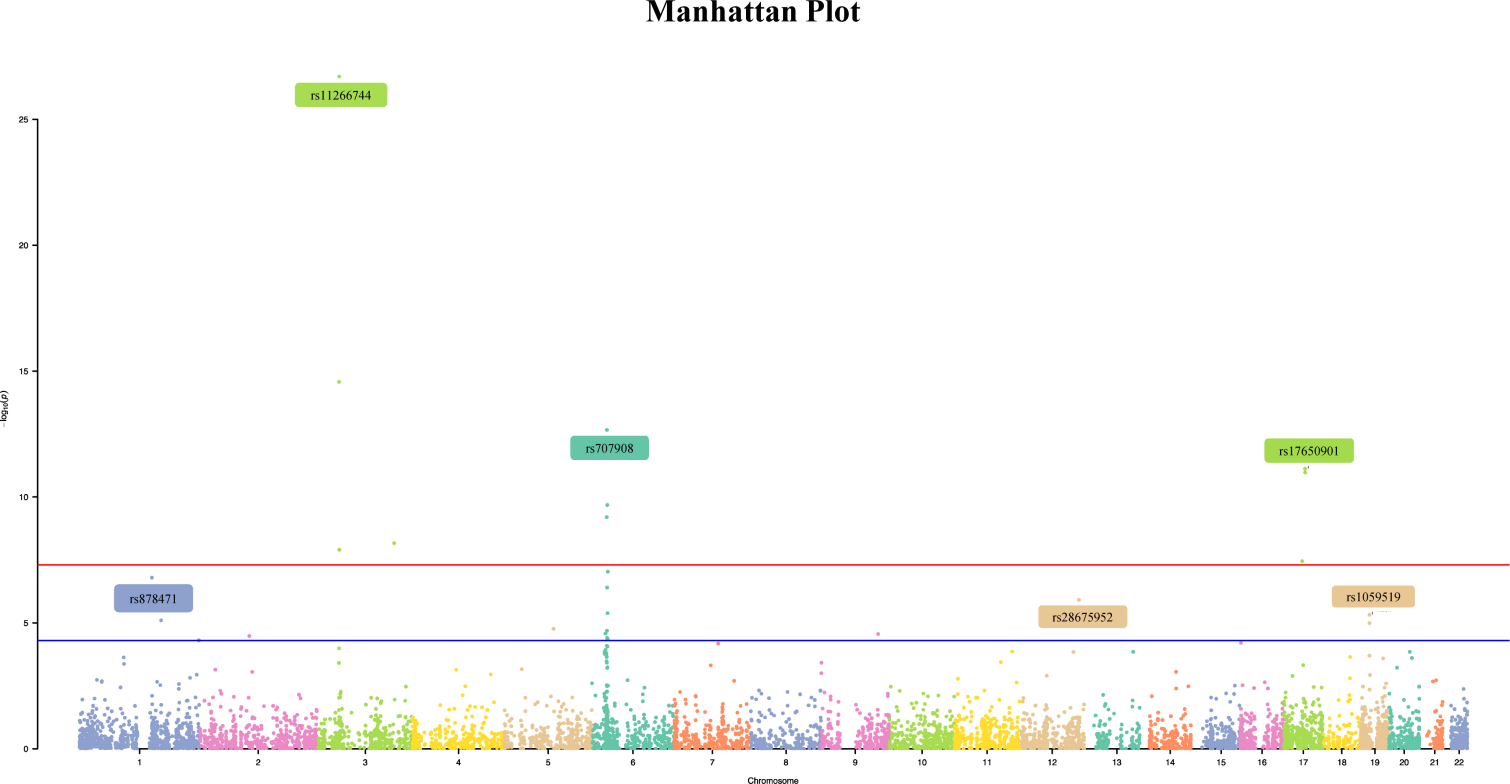
The Manhattan curve of genome-wide identification of m6A-SNPs associated with oral ulcers. The Manhattan plot showed that among the 7490 m6A-SNPs associated with oral ulcers, 11 SNPs reached the genome-wide significance threshold (p<5E- 8), and 30 SNPs reached the genome-wide prompt threshold (p<5E-5).

In this study, 10,599,054 SNPs from the GWAS for oral ulcers and 13,703 m6A-SNPs from the M6Avar database were used to perform the analysis, of which 7,490 m6A-SNPs were identified. (**Figures 1**, **2**). By analyzing the GWAS data, there were 259 m6A-SNPs with high confidence levels among the 7490 m6A-SNPs, and the medium and low levels were 1318 and 5913, respectively. Then the genome-wide threshold p<5.0E-08 (red line) and the recommended threshold p<5.0E-05 (blue line) were used to screen the association between m6A-SNPs and oral ulcers. Finally, rs11266744 (p=2.0E-27) reached the highest significance among the 7490 m6A-SNPs. By using the R package “qqman”, a Manhattan plot containing 7490 M6A-SNPs was generated (**Figure 2**).

### eQTL analysis

m6A-SNPs might participate in gene expression regulation by influencing RNA modification, so eQTL analysis was used to investigate whether they were related to local gene expression levels. A total of 25 m6A-SNPs (p<0.05) associated with oral ulcers showed eQTL signals (**Figure 1**; **Table 1**), and most of them were displayed in various tissues or cells. Among them, the unknown functions of the top 20 (p<0.01) m6A-SNPs in the process of transcriptional regulation were explored. Finally, by using the ENCODE transcription factor CHIP-SEQ dataset, a total of 15 m6A-SNPs were found to change the protein binding or regulatory motifs of local genes in different cell types (**Table 1**) (36).

**Table 1:** Top 20 most significant m6A single nucleotide polymorphisms associated with oral ulcers.

Variant	CHR	Position	Mutation type	*p* value	*Gene*	DEG	eQTL	Proteins bound	Motifs changed	m6A_ID	m6A_Function
rs11266744	3	46408487	Synonymous	2.00E-27	*CCRL2*	Yes	No	No	Yes	m6A_ID_157544	Loss
rs1994492	3	45919154	3’-UTR	2.68E-15	*FYCO1*	Yes	Yes	No	Yes	m6A_ID_157454	Loss
rs707908	6	31270276	Missense	2.17E-13	*HLA-C*	Yes	Yes	No	No	m6A_ID_190475	Loss
rs17650901	17	45962325	5’-UTR	7.54E-12	*MAPT*	Yes	Yes	No	No	m6A_ID_309492	Loss
rs1802036	6	32168252	3’-UTR	2.08E-10	*AGPAT1*	Yes	Yes	Yes	No	m6A_ID_191049	Gain
rs2240803	6	30953180	3’-UTR	6.35E-10	*DPCR1*	Yes	Yes	No	Yes	m6A_ID_190410	Loss
rs6785881	3	160436201	3’-UTR	6.82E-09	*TRIM59*	No	Yes	No	Yes	m6A_ID_165133	Loss
rs11720094	3	46518421	3’-UTR	1.24E-08	*LRRC2*	Yes	Yes	No	Yes	m6A_ID_157583	Gain
rs2305480	17	39905943	Missense	3.54E-08	*GSDMB*	Yes	Yes	No	Yes	m6A_ID_307505	Gain
rs1042136	6	33080851	Missense	9.27E-08	*HLA-DPB1*	Yes	Yes	Yes	No	m6A_ID_191338	Loss
rs878471	1	150575271	3’-UTR	1.60E-07	*MCL1*	Yes	Yes	Yes	Yes	m6A_ID_124174	Loss
rs1046080	6	31628105	Missense	3.97E-07	*PRRC2A*	No	Yes	Yes	Yes	m6A_ID_13969	Loss
rs28675952	12	122715129	3’-UTR	1.22E-06	*HCAR3*	No	Yes	No	Yes	m6A_ID_267011	Loss
rs1059519	19	18386214	Missense	4.72E-06	*GDF15*	No	Yes	Yes	No	m6A_ID_326683	Loss
rs4786	1	169722991	3’-UTR	7.90E-06	*SELE*	Yes	Yes	Yes	Yes	m6A_ID_128464	Gain
rs6512262	19	18391755	3’-UTR	1.02E-05	*LRRC25*	Yes	Yes	No	Yes	m6A_ID_326690	Loss
rs180877323	5	102234749	3’-UTR	1.72E-05	*SLCO4C1*	No	No	No	Yes	m6A_ID_182044	Loss
rs35075694	6	31268757	3’-UTR	2.05E-05	*HLA-C*	Yes	Yes	Yes	Yes	m6A_ID_190474	Gain
rs853678	6	28329536	Missense	2.65E-05	*ZSCAN31*	Yes	Yes	Yes	Yes	m6A_ID_71136	Loss
rs3181371	9	114903290	3’-UTR	2.76E-05	*TNFSF8*	Yes	No	No	Yes	m6A_ID_227454	Loss

### Differential expression analysis

**Figure 3:**
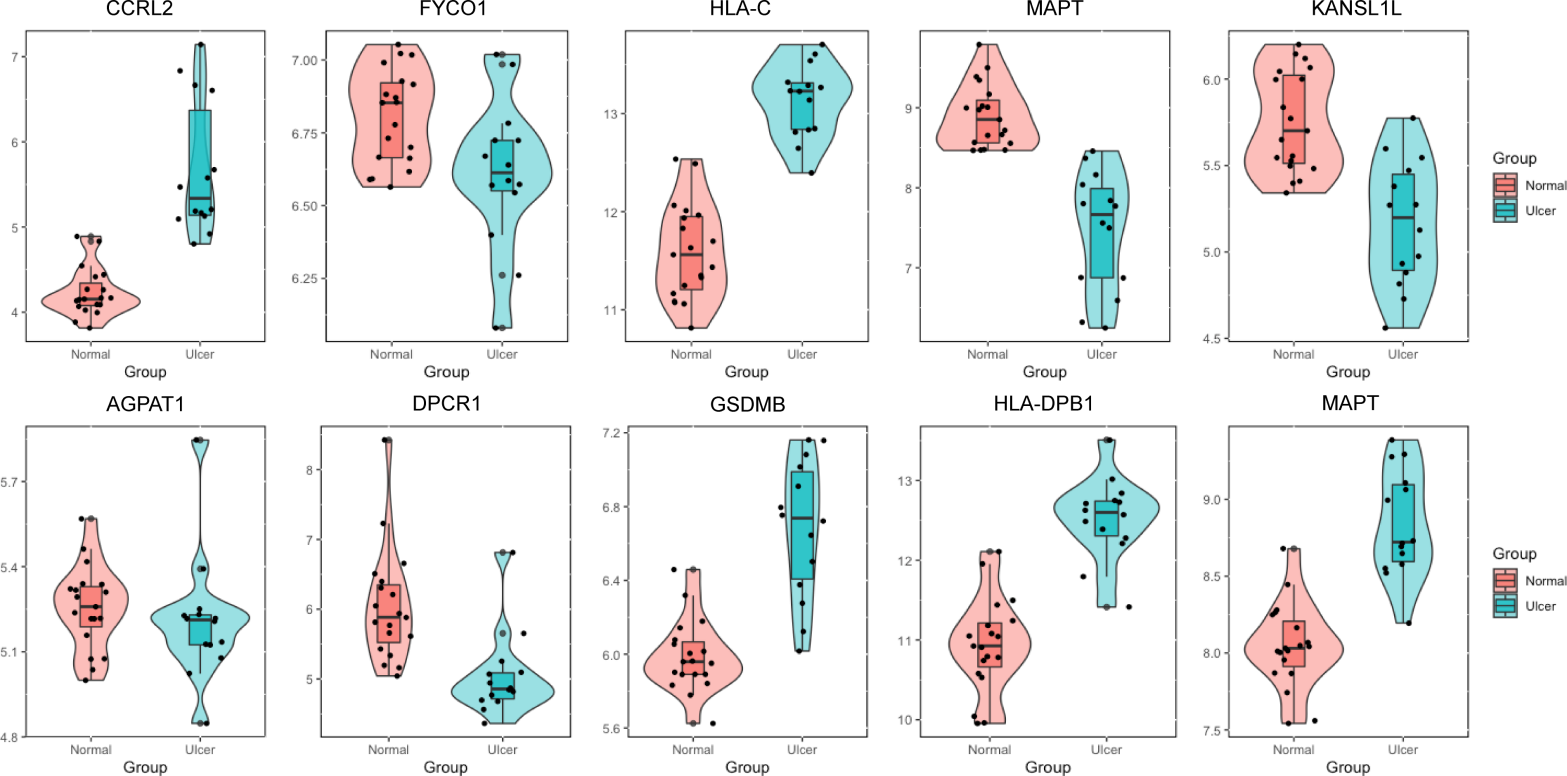
The expression levels of selected genes were shown in oral ulcer patients and healthy controls. The expression of genes such as CCRL2, HLA-C, GSDMB, HLA-DPB1 and MAPT were elevated in patients with oral ulcers.

The appeal analysis found a total of 25 m6A-SNPs showing eQTL signals. In order to analyze the mRNA expression levels of these SNP local genes, we analyzed the difference in gene expression between the ulcer site of patients with recurrent aphthous ulcers and normal control tissues in the GEO database. Through differential expression analysis, 19 m6A-SNPs were detected to form a SNP-gene expression-oral ulcer triad (**Figures 1**, **3**). In **Figure 3**, mRNA levels of differentially expressed genes between oral ulcer patients and normal controls were shown. Among them, the expression of inflammation-related genes such as CCRL2, HLA-C, GSDMB, HLA-DPB1, and MAPT increased in patients with oral ulcers. Based on this, we speculated that the involvement of these m6A-SNPs in the occurrence of oral ulcers might be accomplished by regulating the expression levels of corresponding genes.

## Discussion

m6A modification is one of the most abundant mRNA modifications in higher organisms. It participates in the regulation of biological processes in transcription, including mRNA splicing, translation and stability, and dynamically regulates various physiological and pathological processes (37). In recent years, a large number of studies have focused on the role of RNA modifications in regulating inflammation and anti-inflammatory gene expression. Among them, m6A modification may affect the states of various inflammatory diseases through a variety of mechanisms (38). For example, studies showed that m6A binding protein YTHDF2 could regulate the stability of inflammatory gene mRNA transcripts and participate in the regulation of lipopolysaccharide (LPS)-induced inflammation (39). LPS could damage the oral mucosal barrier and trigger an inflammatory response according to another study, leading to the development of oral ulcers (40).

Genetic variation can affect RNA modification and related biological processes. Studies pointed out that SNPs could affect the RNA secondary structure or the interaction between RNA and protein (31, 41). The important role of SNPs in the development of various diseases has been reported (42, 43), while how m6A-SNPs affect the pathogenesis of oral ulcers has not been confirmed by studies yet. Based on oral ulcer GWAS data and the analysis of the m6A-SNP list in the M6AVar database, we found a large number of related m6A-SNPs and further verified their local gene expression, which would be helpful for better understanding the relationship between m6A-SNPs and the pathogenesis of oral ulcers. Finally, our study shows that the expression of some relevant genes appears to be altered in patients with mouth ulcer. And m6A modifications targeting these genes may contribute to the incidence of oral ulcers. For example, since the CCRL2 protein is associated with leukocyte chemotaxis and the HLA-C protein is a ligand for NK cells, the targeting of the CCRL2 and HLA-C genes would help to reduce the local inflammatory response in the oral mucosa.

**Figure 4:**
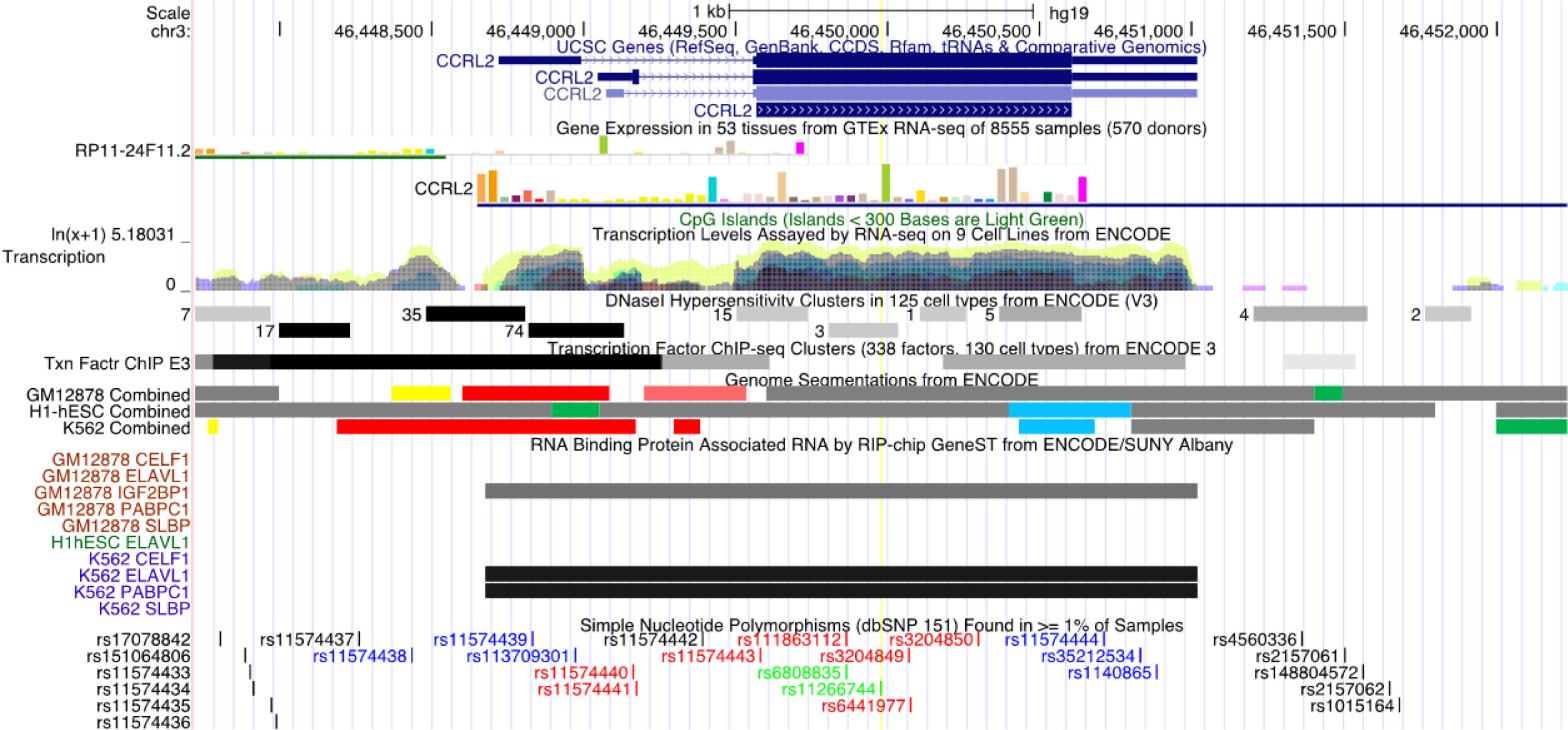
The regional association map of the rs11266744 locus. The SNP rs11266744 was located in the CCRL2 protein-coding region. This region showed very high conservation, transcription level and DNase I hypersensitivity.

**Figure 5:**
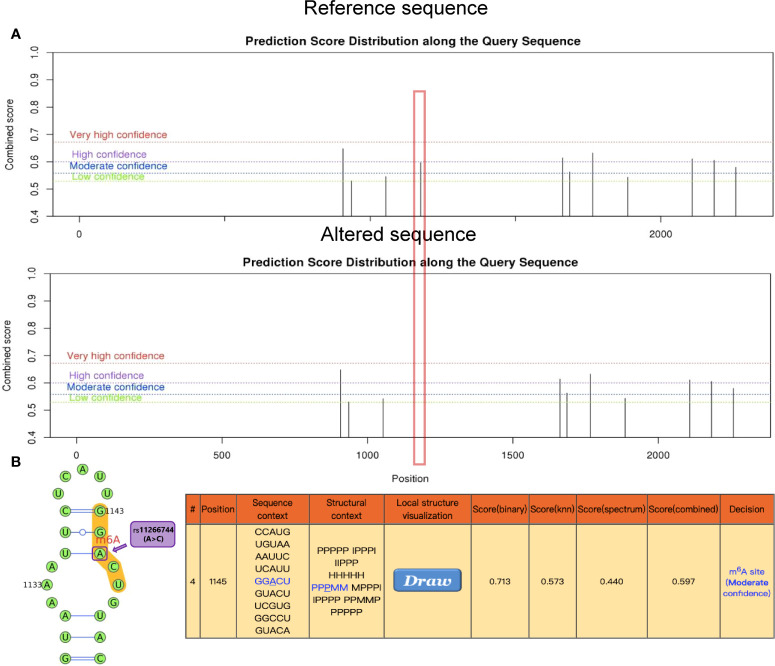
The genomic sequence of a representative CCRL2 transcript (ENST00000399036.4) was used to predict the m6A modification peak on the website (http://www.cuilab.cn/sramp), while secondary structure analysis was enabled. **(A)** The red box indicates the disappearance of the m6A modified peak near rs11266744 after inputting the altered sequence. **(B)** The local secondary structure around mutation site rs11266744 is shown. rs11266744 (A>C) is located in purple at the predicted m6A modification site (red) with medium confidence. In the secondary structure string, H, M, I, B and P refer to hairpin loop, multiple loop, interior loop, bulged loop, and paired residues, respectively.

This study found that the synonymous mutation rs11266744 in the CCRL2 coding region on chromosome 3 could change the motif and approach the DNasel hypersensitive cluster (**Figure 4**). Furthermore, a moderately reliable predicted peak of m6A modification appeared near rs11266744 and would disappear with the change of the input sequence (**Figure 5**). The above evidence indicated that SNP rs11266744 might affect the m6A methylation of mRNA and further change the binding of regulatory motifs, ultimately regulating the expression of gene CCRL2. CCRL2 is a chemokine receptor with seven transmembrane regions, and its only ligand is the non-chemokine chemotactic protein—chemerin (44). When endothelial cells or epithelial cells express CCRL2, the local concentration of chemerin will increase, thereby helping to form a chemotactic gradient for leukocytes expressing CMKLR1 (the functional chemerin receptor) (44). Specifically, the N-terminal of CCRL2 binds chemerin and the C-terminal peptide interacts with CMKLR1, which promotes the aggregation of immune cells expressing CMKLR1 (45, 46). A number of studies proved that CCRL2 was involved in the occurrence and development of inflammatory diseases (47–49). Oral ulcers are characterized by the inflammatory reactions of mucosal epithelial tissues in the oral cavity. During this process, chemokines, chemokine receptors and immune cells will all increase significantly (50, 51). Therefore, the abnormal expression of the gene CCRL2 in mucosal tissues is very likely to trigger inflammation and then induce the occurrence of oral ulcers, and m6A-SNPs play an important role in this process. Further, an analysis of the relationship between loci in CCRL2 and different diseases showed that rs11266744 was most closely associated with oral ulcers (**Supplementary Figure 1**). Based on the above analysis, we speculated that SNP rs11266744 could cause oral ulcers by affecting the expression of the local gene CCRL2.

In addition to inflammation-related genes such as CCRL2, the role of non-inflammation-related genes in oral ulcers is also of interest. rs17650901 (p=7.54E-12) is located on the gene MAPT, which was differentially expressed in the oral ulcer group. The MAPT gene encodes the Tau protein, a microtubule-associated protein, and abnormalities in the Tau protein often cause a number of neurological disorders such as Alzheimer’s disease, frontotemporal dementia and Huntington’s disease (52). Tau proteins promote the polymerization of microtubule proteins into microtubules and maintain their stability, which is associated with normal neuronal function (53). A decrease in Tau protein would reduce the transmission of injurious messages such as pain from the peripheral nervous system to the central nervous system (54). In the oral mucosa, the expression of Tau protein is proved to be associated with cognitive processes such as emotion. Arredondo et al. showed that Tau transcripts were presented at a higher rate in the oral mucosa of cognitively impaired subjects compared to the controls (55). Meanwhile, the expression of MAPT gene was lower in the group of patients with oral ulcers, causing a reduction in Tau protein synthesis. As a result, this reduction reduced the transmission of peripheral injurious stimuli and relieves irritation to the central nervous system, relieving the dysphoria in patients with oral ulcers.

This finding firstly demonstrates that m6A-associated genetic factors are involved in the development of oral ulcers, and there are some shortcomings: (1) lack of publicly available data on relevant proteomic expression profiles, the identified m6A-SNPs have not been validated at the protein expression level. Our study has demonstrated that m6A-SNPs can be involved in the development of oral ulcers by regulating gene expression. More data disclosure of relevant proteomic expression profiles will help to understand the pathogenic mechanisms of m6A-SNPs at the protein expression level; (2) the relationship between the identified m6A-SNP and oral ulcers needs to be verified through case-control studies and electronic replication; (3) whether m6A-SNPs are involved in the development of oral ulcers by affecting gene expression has not been clarified, and how it is involved in the pathogenesis of oral ulcers also remains to be verified.

## Conclusion

In summary, this study revealed for the first time that m6A modification may be involved in the pathogenesis of oral ulcers. By bioinformatic analysis, the m6A-SNP rs11266744 was identified to be associated with the m6A modification of the CCRL2 gene. m6A modification affected would be involved in the development of intraoral inflammation by inducing the aggregation of leukocytes expressing CMKLR1.

## Data availability statement

The original contributions presented in the study are included in the article/**supplementary material**. Further inquiries can be directed to the corresponding author.

## Ethics statement

Written informed consent was obtained from the individual(s) for the publication of any potentially identifiable images or data included in this article.

## Author contributions

ML conceived the study. ZW and WL designed the experiments. ZW, WL, and ML performed the experiments. ZW and WL analyzed the data. QY helped with the statistical analysis of the data. ZW and ML wrote the manuscript. All authors contributed to the article and approved the submitted version.

## Funding

National Natural Science Foundation of China (NSFC 81900967), Sichuan Science and Technology Program (2020JDRC0023).

## Acknowledgments

We appreciate this opportunity to cooperate, and we are grateful for the support from National Natural Science Foundation of China (NSFC 81900967), Sichuan Science and Technology Program (2020JDRC0023) throughout the study.

## Conflict of interest

The authors declare that the research was conducted in the absence of any commercial or financial relationships that could be construed as a potential conflict of interest.

## Publisher’s note

All claims expressed in this article are solely those of the authors and do not necessarily represent those of their affiliated organizations, or those of the publisher, the editors and the reviewers. Any product that may be evaluated in this article, or claim that may be made by its manufacturer, is not guaranteed or endorsed by the publisher.
