# Supplementary material for: A genome-wide association analysis: m6A-SNP related to the onset of oral ulcers
Source: Front Immunol. 2022 Jul 25;13:931408. doi: 10.3389/fimmu.2022.931408 (PMC9357892; doi:10.3389/fimmu.2022.931408)
Supplement: Supplementary file 1 [file DataSheet_1.docx]

Supplementary Material

**
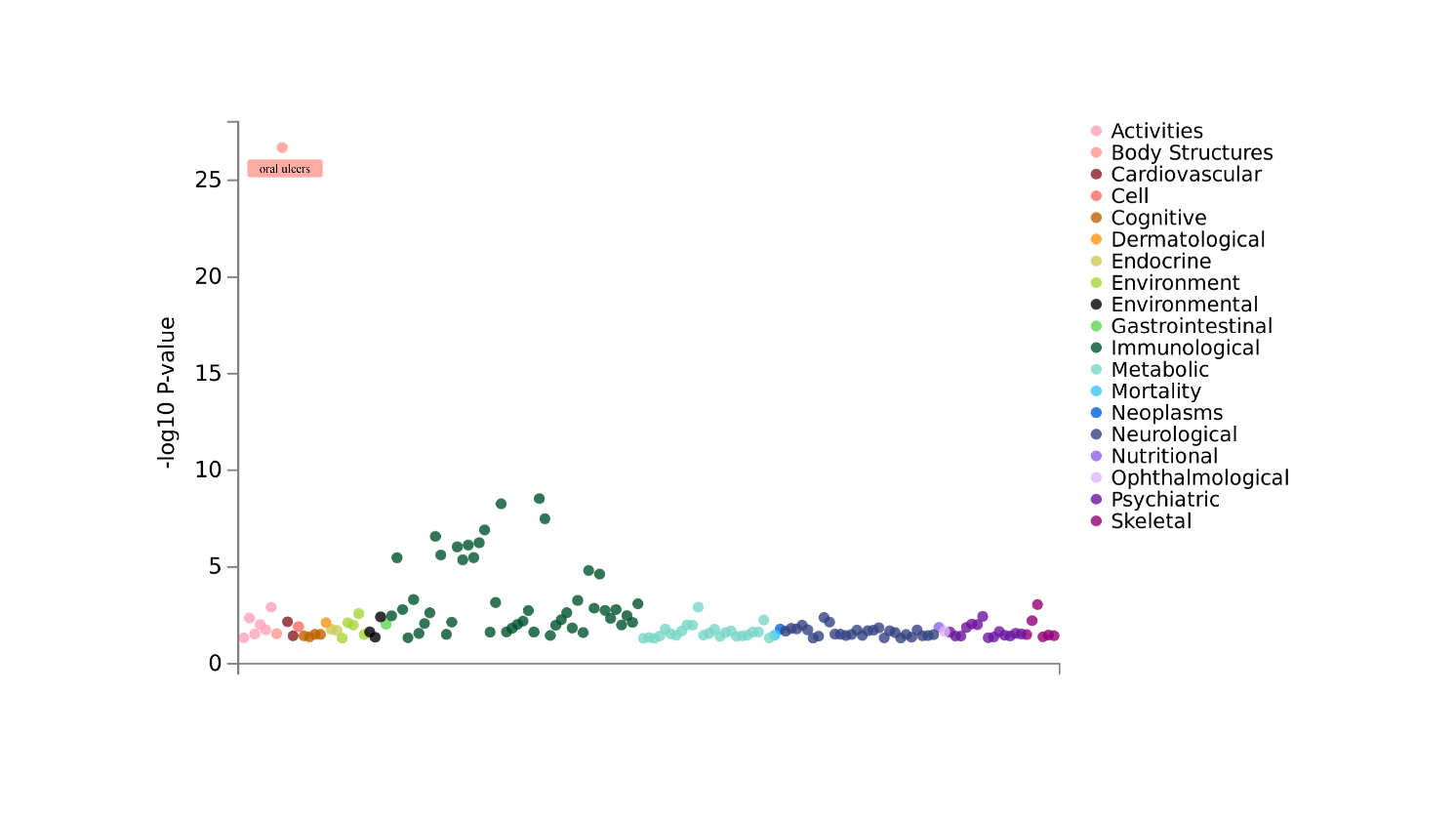
**

**Supplementary Figure 1.** The association between CCRL2 gene locus rs11266744 and different diseases or different phenotypes was expressed by the size of P value, among which oral ulcers were the most correlated
